# Asymmetries in Ground Reaction Forces During Turns by Elite Slalom Alpine Skiers Are Not Related to Asymmetries in Muscular Strength

**DOI:** 10.3389/fphys.2021.577698

**Published:** 2021-03-30

**Authors:** Jan Ogrin, Nejc Šarabon, Mads Kjær Madsen, Uwe Kersting, Hans-Christer Holmberg, Matej Supej

**Affiliations:** ^1^Faculty of Sport, University of Ljubljana, Ljubljana, Slovenia; ^2^Faculty of Health Sciences, University of Primorska, Izola, Slovenia; ^3^S2P, Science to Practice, Ltd., Laboratory for Motor Control and Motor Behavior, Ljubljana, Slovenia; ^4^Department of Health Science and Technology, Sport Sciences – Performance and Technology, Aalborg University, Aalborg, Denmark; ^5^Institute of Biomechanics and Orthopedics, German Sport University Cologne, Cologne, Germany; ^6^Department of Physiology and Pharmacology, Biomedicum C5, Karolinska Institute, Stockholm, Sweden; ^7^China Institute of Sport and Health Science, Beijing Sport University, Beijing, China; ^8^Swedish Winter Sports Research Centre, Mid Sweden University, Östersund, Sweden

**Keywords:** biomechanics, alpine skiing, inertial suit, GNSS-global navigation satellite system, GPS-global positional system, pressure insoles, force plate

## Abstract

The ground reaction forces (GRF) associated with competitive alpine skiing, which are relatively large, might be asymmetric during left and right turns due to asymmetries in the strength of the legs and torso and the present investigation was designed to evaluate this possibility. While skiing a symmetrical, 20-gate slalom course, the asymmetries of 9 elite alpine skiers were calculated on the basis of measurements provided by inertial motion units (IMU), a Global Navigation Satellite System and pressure insoles. In addition, specialized dynamometers were utilized to assess potential asymmetry in the strength of their legs and torso in the laboratory. In total, seven variables related to GRF were assessed on-snow and eight related to strength of the legs and torso in the laboratory. The asymmetries in these parameters between left and right turns on snow were expressed in terms of the symmetry (SI) and Jaccard indices (JI), while the asymmetries between the left and right sides of the body in the case of the laboratory measurements were expressed as the SIs. The three hypotheses to be tested were examined using multivariable regression models. Our findings resulted in rejection of all three hypotheses: The asymmetries in total GRF (H1), as well as in the GRF acting on the inside and outside legs (H2) and on the rear- and forefeet GRF (H3) during left and right turns were not associated with asymmetries in parameters related to muscular strength. Nevertheless, this group of elite slalom skiers exhibited significant asymmetry between their right and left legs with respect to MVC during ankle flexion (0.53 ± 0.06 versus 0.60 ± 0.07 Nm/kg, respectively) and hip extension (2.68 ± 0.39 versus 2.17 ± 0.26 Nm/kg), as well as with respect to the GRFs on the inside leg while skiing (66.8 ± 7.39 versus 76.0 ± 10.0 %BW). As indicated by the JI values, there were also large asymmetries related to GRF as measured by pressure insoles (range: 42.7–56.0%). In conclusion, inter-limb asymmetries in GRFs during elite alpine skiing are not related to corresponding asymmetries in muscular strength. Although our elite athletes exhibited relatively small inter-limb asymmetries in strength, their asymmetries in GRF on-snow were relatively large.

## Introduction

Alpine ski racing is an extremely complex and highly competitive sport, involving numerous physical, technical and tactical challenges. The difference between the first and second finishers in a race is often only hundredths of a second (Hébert-Losier et al., 2014). Accordingly, small differences in a variety of factors can exert considerable influence on the successful performance of elite alpine ski racers.

Although several studies have focused on determinants of sectional or instantaneous performance (Hébert-Losier et al., 2014), there is still no deeper understanding of the influence of biomechanical, anatomical variables or physical preparation (Spörri et al., 2012; Supej and Holmberg, 2019). In this context, vertical jump efficiency has been shown to be a good predictor of slalom performance (Strojnik and Dolenc, 2009), although at the same time Schobersberger et al. (2021) demonstrated recently that neither maximal aerobic capacity nor maximal power output were significantly correlated to competitive performance, as indicated by FIS ranking. These findings reinforce the proposal that there is no single determinant of competitive performance in this complex sport.

The risk of injury associated with alpine skiing is relatively high (Haaland et al., 2016) and recent research has focused on determining why and, at the same time, reducing this risk (Bere et al., 2014; Müller et al., 2016; Spörri et al., 2017). The handful of reports on asymmetries in alpine skiing that have appeared so far have been concerned primarily with the effects of such asymmetries on injuries to the anterior cruciate ligament of the knee (Bujas et al., 2012; Jordan et al., 2015a, 2017; Steidl-Muller et al., 2018). A 10% asymmetry in the strength of the legs is generally considered to be the threshold for a safe return to alpine skiing following injury. Interestingly, healthy, uninjured younger skiers often exhibit more pronounced asymmetry in leg extension strength during their phases of rapid growth, suggesting that a different threshold might be more appropriate for this particular subgroup (Steidl-Muller et al., 2018). However, that same investigation also found that differences between the right and left legs with respect to extension strength considerably increase the risk of injury for young ski racers.

The relationship between asymmetries with incidence and risk of injuries in connection with other sports has also been receiving attention. For both athletes and individuals who not participate in sports, inter-limb asymmetries > 15% are associated with an elevated risk of injury (Barber et al., 1990). Although it is reasonable to conclude that reducing such asymmetry can help avoid injuries, the actual effect of interventions designed for this purpose remains unclear.

Recently, Bell et al. (2014) reported that more pronounced inter-leg asymmetry (>10%) in power production during the push-off phase of vertical jumping is associated with 20–25% lower jump height, as well as less favorable changes in direction. Moreover, a systematic review exploring the relationship between asymmetries and performance describes widely discrepant findings, with negative associations in some cases (e.g., jumping and kicking), no associations in others (e.g., sprinting), and occasionally even a positive relationship (e.g., cycling) (Bishop et al., 2018; Maloney, 2019). Such relationships may be dependent on the sport under consideration, as well as the age and sex of the athletes.

On-snow training, off-snow resistance training and conditioning of elite alpine skiers have received relatively little attention from sports researchers (Gilgien et al., 2018). Indeed, our search of the relevant scientific literature revealed only one study dealing with the laterality of ground reaction forces (GRF) in connection with slalom skiing (Vaverka and Vodickova, 2010) and one other case study demonstrating that an inertial motion tracker placed on the pelvis can detect lateral asymmetries during giant slalom skiing (Yu et al., 2016). More recently, Supej et al. (2020) found that asymmetries in technique and GRF were associated with asymmetries in performance. More specifically, asymmetries in GRF acting on the outside leg in combination with the shank angle influenced the asymmetries in turning radius.

To our knowledge, kinematic and/or kinetic data have not yet been employed to search for potential links between asymmetries in muscular strength and asymmetries on-snow. This lack of research is especially disturbing since the GRFs associated with alpine skiing are large (Supej and Holmberg, 2019) and fundamental motor asymmetries may potentially exert considerable influence on both skiing performance and safety.

The large GRFs associated with alpine skiing require considerable muscular strength for effective turning. Consequently, lateral asymmetry by and the strength of the legs and trunk might result in differences between left and right turns and, thereby, different patterns of GRFs. Accordingly, this study was designed to determine whether asymmetries in basic muscular strength are related to the GRFs encountered during elite slalom skiing. In this context and based on expert deconstruction of skiing as a motor task, the following three hypotheses were tested:

H1: Asymmetries in the GRFs associated with left and right turns are related to asymmetries between the left and right sides of the body with respect to at least one of the following parameters related to muscular strength: maximal force during a countermovement jump and/or the peak torque associated with maximal voluntary contraction (MVC) during knee extension and flexion and/or during hip extension.

H2: Asymmetries between left and right turns regarding the GRF acting on the inside and/or outside leg are related to asymmetries between the left and right sides of the body with respect to at least one of the following parameters related to muscular strength: maximal force during a countermovement jump and/or peak MVC torque during knee extension and/or flexion, during hip extension and/or abduction, and/or during lateral trunk flexion.

H3: Asymmetries between left and right turns with respect to the GRF acting on the rear- and forefoot of the inside and outside legs are related to asymmetries between the left and right sides of the body regarding peak MVC torque during ankle extension and/or flexion.

## Materials and Methods

### Measurements and Collection of Data

#### Skiing Tests

During three consecutive days, data on 9 elite male European Cup slalom skiers (age: 22.7 ± 3.4 y; height: 181.8 ± 6.9 cm; weight, 82.2 ± 5.6 kg; current SL FIS points: 24.9 ± 18.6; means ± SD), five with right and four left leg preference (assessed on the basis of leg preference in connection with testing maximal height of a single-leg jump), were collected on a groomed slope with a mean inclination of 16° and <1° tilting to either side on a glacier. The snow was icy and hard and the temperature between −2 and 0°C. The 20-gate corridor course, with two symmetrical (mirrored) slalom courses (see Supplementary Figure 1), was set up using a high-resolution geodetic global satellite navigation system (Leica Geosystems 1200, Leica Geosystems AG, Heerbrugg, Switzerland) and its accompanying “Stakeout” program to ensure a constant distance of 12 and 4-m offset between the gates. The total length of the course from the start to the finish was approximately 250 m. These two courses were prepared professionally following each of the 12 individual runs on any given day. This study was pre-approved by the National Medical Ethics Committee (approval no. 0120-99/2018/5) and informed written consent to participate obtained from each subject.

**FIGURE 1:**
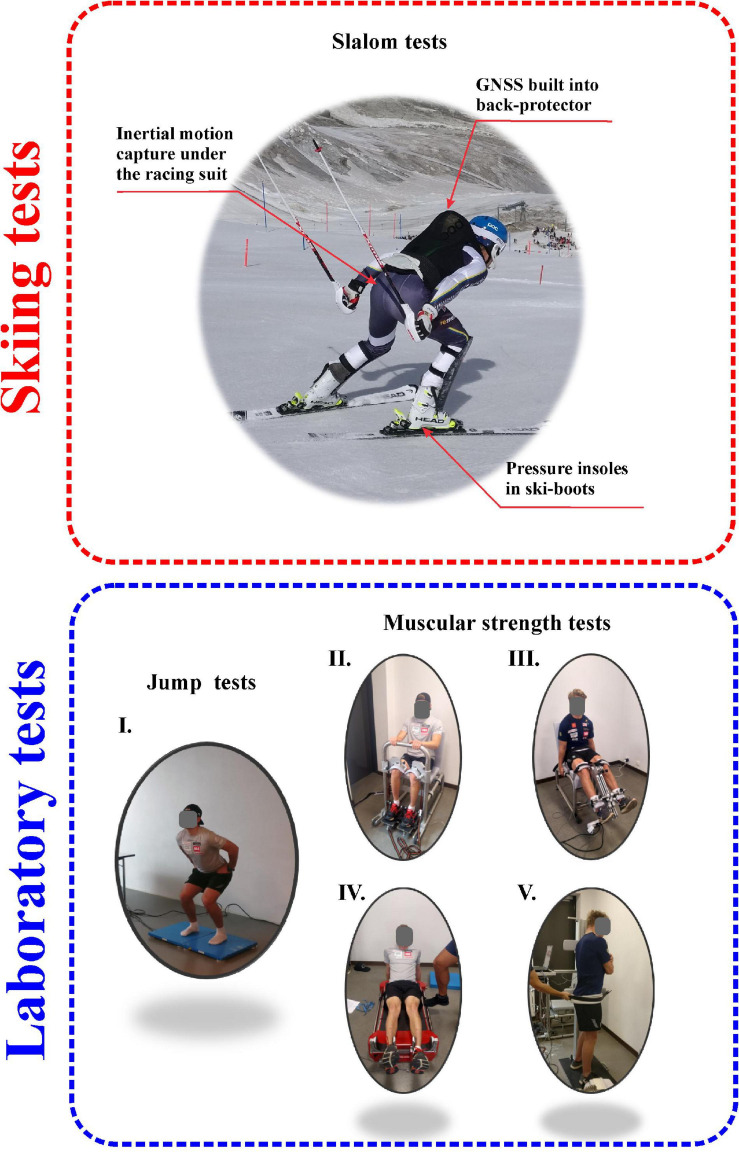
Setups for the skiing measurements on snow (top) and laboratory tests (bottom): (I) countermovement jump on a bilateral force platform, (II) ankle dynamometry, (III) knee dynamometry, (IV) hip dynamometry, and (V) trunk dynamometry.

Each subject skied this corridor course four times and all trials were used in our calculations. The side from which the first run was started was pre-selected randomly (and the skier informed about this assignment at the start), with subsequent starts alternating between the left and right sides. Prior to each and every run, the course was smoothed by coaches and members of the research team in order to provide nearly ideal conditions. Prior to each run, the skier was asked to perform three explosive squats, followed by three hits with the right ski to the ground, in order to synchronize the measuring instruments. The three-dimensional motion of the entire body was monitored with a full-body inertial measurement system in combination with a high-frequency Real Time Kinematics Global Navigation Satellite System (RTK GNSS) (Figure 1), in a manner similar to previous studies (Krüger and Edelmann-Nusser, 2010; Supej, 2010). The only difference was that here a more recent inertial system MVN Biomech. 2018.2 (Xsens Technologies B.V., Enschede, Netherlands) recording data at 240 Hz and a more advanced/new generation GNSS antenna Leica Zeno GG04 plus (Leica Geosystems, Heerbrugg, Switzerland) with a sampling frequency of 20 Hz were used.

The inertial sensors were embedded and placed under the skier’s racing suit, while the GNSS smart antenna (weight: 0.8 kg, height: 7.1 cm, diameter: 8.6 cm) was placed on top of a modified back-protector vest (Spine VPD 2.0, POC, Stockholm, Sweden) to ensure optimal satellite visibility in the vicinity of the shoulders and posterior neckline (Figure 1). The receiver (Conker NS6, Conker, Takeley, England) was placed in a pocket of the back-protector vest located on the front of the trunk and the two devices communicated via Bluetooth. To allow merging of the trajectories surveyed by RTK GNSS with inertial data on full-body motion, the position of the antenna relative to the cervical (C7) and thoracic vertebra (T12) was measured. GRFs were monitored at a sampling frequency of 100 Hz by pressure insoles (Loadsol, Novel GmbH, Münich, Germany) that allowed separate determination of the forces on the left and right feet and fore- and rear-foot, as well as total overall force (Figure 1). To ensure appropriate synchronization, while skiing the subjects were also recorded at 50 Hz with a high-resolution JVC camcorder (GC-PX100, The Japan Victor Company Ltd., Yokohama, Japan).

#### Laboratory Tests

On the day following these skiing runs, counter-movement jump performance and maximal isometric strength of the selected muscle groups was tested. It was ensured that there was sufficient time for rest between the measurements in the laboratory and on-snow. Maximal isometric voluntary torque during bilateral plantar flexion and dorsal flexion was tested on an isometric ankle dynamometer (S2P Ltd., Ljubljana, Slovenia) adjusted to ensure proper positioning of each subject (upright trunk, 90° angles at the hips, knees and ankles). A metal brake above each knee and a rigid strap above each foot ensured isometric conditions. Each of the two foot pedals was linked firmly to a strain gauge (Z6FC3/200kg, Hottinger Baldwin Messtechnik GmbH, Darmstadt, Germany). For determination of maximal voluntary torque during bilateral flexion and extension of the knee, the subject was seated (with 90° and 60° flexion at the hips and knees, respectively) on an isometric knee dynamometer (S2P Ltd., Ljubljana, Slovenia) with tight straps across the pelvis, above the knees and behind the distal leg to ensure fixation. This positioning of the joints was chosen to allow the knee flexors/extensors to develop maximal knee torque (Brughelli and Cronin, 2007; Brughelli et al., 2010). This testing procedure has been reported to yield highly reproducible results (Sarabon et al., 2013).

The maximal strength of muscle groups acting at the hip during bilateral abduction and unilateral flexion (supine position) was evaluated employing a multi-directional hip isometric dynamometer (Muscle Board, S2P Ltd., Ljubljana, Slovenia). During these tests, performed both in the prone (hip extension) and supine (hip abduction) positions, the pelvis was fixated with tight non-elastic straps. The lateral trunk flexors were tested in the upright position utilizing the dynamometric function of a multi-modal system for testing neuromuscular functions of the trunk (TNC system, S2P Ltd., Ljubljana, Slovenia).

In all cases, testing of maximal voluntary strength followed the same protocol. After the task was explained to the subject, the equipment fitted and appropriate/firm fixation of body segments ensured, the subject first carried out one repetition each at approximately 50, 75, and 90% of self-estimated maximal voluntary effort to become accustomed to the set-up. Thereafter, he performed three MVCs (pressing against the support with as much force as possible for 3 s), separated by 60 s to avoid fatigue and with verbal encouragement. The signals were acquired at 1,000 Hz (NI USB-6009; National Instruments Corp., Austin, TX, United States) and the 1-s period around the peak of the torque-time curve analyzed. The mean value of the three repetitions was used for statistical analysis.

GRFs during bilateral counter-movement jump were determined with a bilateral force plate (2x 9260AA6, Kistler Instrumente AG, Winterthur, Switzerland). Each participant was instructed to perform a rapid counter-movement (so that the angle of the knee reached 90°) and couple this explosively to the push-off to jump as high as possible. Jumps that were not explosive, i.e., the eccentric and concentric phases were not coupled explosively and/or the heels not lifted off the force plate during the downward movement, were rejected and the trial repeated. The subject was asked to aim for maximal performance during three repetitions while adhering to the following instructions: (i) counter-movement associated with the counter-movement jump should be performed quickly and dynamically; and (ii) the hands should be on the hips at all times and the feet flat and in contact with the force plate during push-off.

### Computation of Independent and Dependent Variables

#### Independent Variables

*The values were calculated using the following symmetry index (SI), which is similar to the one developed by* Bishop et al. (2016):

$$S\,I\,\left(L,R\right)=1-\frac{\left|L-R\right|}{L\,R}.$$

where *L* represents the measurement for the left side and *R* the right measurement. The means of the 1-s maxima in the three successful trials (derived from the torque/time curve in the case of the isometric dynamometer and from the force/time curve for the force plate) were employed. In a similar manner, the SI was computed using the mean percentages of body weight supported by the left and right legs, obtained from the force plate signals during the quiet stances, as in the work of Sarabon and Rosker (2013). In this manner the level of fundamental motor asymmetry was derived from the following asymmetry indicating variables:

– the SI for the maximal force during the counter-movement jump;
– the SI for the maximal voluntary contraction (MVC) associated with flexion and extension of the knee and ankle;
– the SI for the MVC associated with hip flexion and abduction;
– the SI for the MVC during lateral flexion of the trunk.

A high SI percentage means better symmetry between the left and right side measurement.

#### Dependent Variables

The kinematic data supplied by the MVN Biomech were exported into Matlab R2016b (Mathworks, Natick, MA, United States) for further processing. To match their sampling frequency to that of the inertial sensors, the RTK GNSS and force data were converted to 240 Hz by cubic spline interpolation. The RTK GNSS, full-body inertial motion and video data were synchronized using the lowest point during the final explosive squat movement. Force and kinematic data were synchronized by matching the peak foot acceleration with the maximal GRF during the three hits with the ski on the snow before the start of testing.

Following synchronization, all data were filtered with the Rauch-Tung-Striebel algorithm (Rauch et al., 1965), which uses two unscented Kalman filters running forward and backward in time to achieve fixed-interval offline smoothing of the estimated signals, as described previously (Supej, 2010). The RTK GNSS and measurements of full-body inertial motion were merged employing the GNSS data as a global reference coordinate system. These merged data was then exported into the Visual 3D v6 software (C-Motion Inc., Germantown, MD, United States), which computed the trajectory of the center-of-mass (CoM) and acceleration of the skier, as well as the trajectory of the mid-point of the ankle (arithmetic mean of the positions of the ankle joints), after which the values obtained were exported back into formats compatible with Matlab, in a manner similar to that described previously (Zorko et al., 2015). The sum of the CoM and gravitational acceleration was used to estimate the total GRFs acting on the skier (Supej and Holmberg, 2010). The variables of GRF obtained from both the kinematic system and pressure insoles had also been normalized to the overall GRF to facilitate analysis and are presented as percentages of body weight (% BW). It should be noted that measurements of forces by pressure insoles have been reported to be too inaccurate for monitoring the magnitude of the GRF (Stricker et al., 2010). However, since it can be assumed that the GRF values indicated by pressure insoles are subject to approximately the same level and type of bias in the case of the left and right legs, these values can be used for calculating symmetry indices, which only reflect relative differences.

For all variables, mean curves and standard deviations were calculated and diagrams representing turn-cycle characteristics were created using the definitions of turn phases described by Müller et al. (1998), while the beginning and end of each turn were defined according to Supej et al. (2003). For this latter purpose, a 3D terrain model of the slope was constructed and the turns analyzed were divided into initiation, steering and completion phases, as previously described in greater detail (Supej and Holmberg, 2010).

The largest GRFs associated with slalom occurs during the steering phase, when the skier is actively turning, whereas during the initiation and completion phases these forces are significantly lower (Supej and Holmberg, 2010; Supej et al., 2015). Therefore, the SI reflecting the degree of asymmetry in the GRF was determined during the steering phase. In addition, the Jaccard index (JI) (Jaccard, 1901) or intersection over union (IOU), a standard algorithmic estimation of the accuracy with which an object is detected by a computer vision, was calculated on the basis of the shapes of the left (*Lg*) and right (*Rg*) turns. Definition of these shapes was based on a normalized time-scale from the start to finish of the turn, with upper and lower limits one standard deviation above or below the average curve, respectively. The average curve and corresponding standard deviation for *Lg* and *Rg* was calculated from all left turns and all right turns, respectively. The Jaccard index (JI) was then calculated from these two geometric shapes *Lg* and *Rg* as follows:

$$J\,I\,\left(L\,g,R\,g\right)=\frac{p\,\left(L\,g\cap R\,g\right)}{p\,\left(L\,g\cup R\,g\right)},$$

where p represents the area enclosed by the geometric shapes (*Lg*∩*Rg* or *Lg*∪*Rg*).

In contrast to the situation with SI, not only the mean values for JI, but also the standard deviations are utilized to calculate asymmetries between sets of data and, therefore, JI values provide a better indication of asymmetry. Moreover, there is a bijective relationship between geometrical matching of Lg and Rg and matching of the means and standard deviations of the values for these datasets, which both determine the same two-dimensional shapes. In other words, when there is a good match between the mean values and standard deviations for the two sets, there is a good geometrical match (i.e., JI is close to 1) between the two two-dimensional shapes determined by these same two sets, and vice versa.

From a practical point of view, the maximal or average GFR during a slalom turn captures very few aspects of this action. Maximal GRF can often be generated by shocks or transient vibrations that have no major impact on skiing (Supej et al., 2018). Moreover, not only do the GRFs during the weight transfer and steering phases differ, the magnitude of the GRF within the steering phase also varies significantly (Supej and Holmberg, 2010; Supej et al., 2015). Therefore, averaging the GRF for the entire steering phase results in loss of important information. By determining the JI for the “steering phase,” this problem can be avoided and a deeper understanding of “active” turning achieved.

Combining SI and JI provided a measure of the level of GRF asymmetry, in particular:

– the SI and JI for GRFs acting on the inside or outside of the fore-, rear- or entire foot during the steering phase (measured by the pressure insoles);

– the SI and JI for the overall GRF during the steering phase (computed from the kinematic data).

### Statistical Analyses

All values presented are means and standard deviations. The Shapiro-Wilk test was applied to determine whether the distribution of these values was normal and, when necessary, the Box-Cox power transformation performed to achieve normality. The data on turns were examined for potential outliers (1.5 Inter-Quartile Range method of outlier detection was applied), which were excluded from analysis. For all possible combinations of the independent (at most two) and dependent variables, multivariable linear regression was performed. Initial *p*-values below 0.05 obtained by applying the *F*-test to regression models were adjusted using a Bonferroni- like correction (Curtin and Schulz, 1998). The power of differences indicated as being statistically significant (*p* < 0.05) was tested using G^∗^Power (Faul et al., 2009) and only differences with a power greater than 0.8 were finally considered to be significant. All statistical analyses were performed in the Matlab software (Prentice-Hall, Inc., Upper Saddle River, NJ, United States).

## Results

### The Symmetry and Jaccard Indices (SI and JI)

The mean symmetry indices (SI) for the independent variables determined in the laboratory ranged from ∼89% (for peak MVC torque during hip extension) to ∼98% (peak MVC torque during hip abduction) (Table 1), while the corresponding mean SI for the dependent variables during the steering phase of the turn while skiing ranged from ∼85% (GRFs on the rear-foot of the inside leg) to ∼98% (total GRF). The mean Jaccard Index (JI) for the dependent variables during this steering ranged from ∼43% (for the GRF on the fore-foot of the inside leg) to ∼71% (total GRF) (Table 2).

**TABLE 1:** The values (means ± standard deviations, statistical comparison of the right and left legs) of the parameters measured and corresponding motor symmetry indices (independent variables).

**Variable**	**Left leg/side**	**Right leg/side**	***p*-value**	**Cohen’s d**	**SI (%)**
**Countermovement jump**					
Maximal force (N/kg)	12.2 ± 1.20	13.3 ± 1.64	0.12	0.61	94.0 ± 3.14
**Maximal voluntary contraction**					
Ankle flexion (Nm/kg)	0.53 ± 0.06	0.60 ± 0.07	0.04	0.84	93.5 ± 3.74
Ankle extension (Nm/kg)	1.86 ± 0.27	1.93 ± 0.32	0.62	0.19	95.6 ± 2.60
Knee flexion (Nm/kg)	1.81 ± 0.24	1.83 ± 0.16	0.84	0.07	95.6 ± 3.80
Knee extension (Nm/kg)	2.75 ± 0.47	2.69 ± 0.42	0.80	0.10	95.5 ± 2.60
Hip extension (Nm/kg)	2.68 ± 0.39	2.17 ± 0.26	0.01	1.14	89.5 ± 6.33
Hip abduction (Nm/kg)	1.77 ± 0.28	1.87 ± 0.36	0.52	0.02	97.7 ± 1.88
Trunk lateral flexion (Nm/kg)	6.68 ± 1.30	7.36 ± 1.18	0.26	0.43	92.7 ± 5.60

**SI, symmetry index.**

**TABLE 2:** The values (means ± standard deviations, statistical comparison of the right and left legs) of the ground reaction forces (GRF) measured and corresponding symmetry and Jaccard indices (dependent variables).

**Variable**	**Left turn**	**Right turn**	***p*-value**	**Cohen’s d**	**SI** (**%**)	**JI** (**%**)
**Entire foot—pressure insoles**						
GRF outside leg (% BW)	126.2 ± 19.2	113.6 ± 21.55	0.21	0.48	92.9 ± 4.74	56.1 ± 18.9
GRF inside leg (% BW)	66.8 ± 7.39	76.0 ± 10.0	0.04	0.83	91.4 ± 5.72	56.0 ± 19.6
**Fore-foot—pressure insoles**						
GRF outside leg (% BW)	62.5 ± 20.6	58.7 ± 26.85	0.75	0.12	88.4 ± 8.35	47.2 ± 21.6
GRF inside leg (% BW)	27.4 ± 9.20	37.8 ± 17.6	0.14	0.58	85.1 ± 10.1	42.7 ± 23.2
**Rear-foot—pressure insoles**						
GRF outside leg (% BW)	63.8 ± 7.69	54.9 ± 16.6	0.17	0.54	87.8 ± 7.33	51.6 ± 14.4
GRF inside leg (% BW)	39.4 ± 9.13	38.25 ± 15.8	0.85	0.07	85.8 ± 15.2	52.9 ± 23.2
**Approximation from CoM**						
Overall GRF (% BW)	287.5 ± 26.3	283.7 ± 17.5	0.72	0.13	98.2 ± 1.11	71.3 ± 2.70

**CoM, center of mass; BW, body weight; SI, symmetry index; JI, Jaccard index.**

**FIGURE 2:**
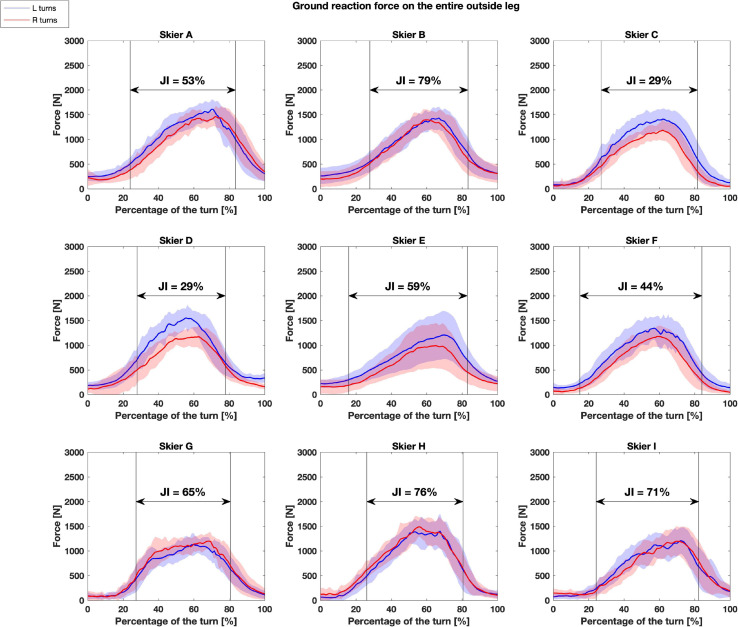
Evolution of the ground reaction forces (GRFs) on the entire outside foot during left (blue) and right (red) turns for all nine elite alpine skiers **(A-I)** in order to compare the Jaccard Index (JI) during the steering phase. The vertical lines indicate the start and end of the turn and the steering phase is marked with a horizontal double-arrow.

The time-courses of the GRFs on the entire outside foot during left and right turns and the corresponding JI during the steering phase (ranging from 29 to 79%) are shown for all 9 skiers in Figure 1. For four of these skiers, these two variables were well matched, while the other 5 demonstrated asymmetry in GRFs on the outside leg between 29 and 59% (skiers A, C, D, E, and F in Figure 2). Of these latter 5, 4 had a right leg preference and larger mean overall GRFs during the steering phase of left turns, when the right leg was outside.

### Multi-Variable Regression

To test our three hypotheses, linear multivariable regression models were applied. In the case of hypothesis H1, four independent variables and two dependent variables were included and 20 linear regression models tested. For H2 and H3, 124 and 24 models were tested, respectively. In each case, depending on the number of models, the threshold *p*-value obtained was subjected to Bonferroni adjustment. All models that yielded a *p*-value greater than the adjusted threshold were discarded, as were all models for which R2 was less than 0.7. These restrictions led to all of the models being discarded and thus to all of the hypotheses being rejected. Although none of the models was associated with a significant *p*-value, Tables 3–5 present the findings with models that included a single potential predictor. Models including more than one potential predictor are not documented because of their very large total number (80).

**TABLE 3:** Triplets (*p*-value, coefficient in the linear regression model, *R*^2^) for each pair: independent (laboratory) variable (rows) and dependent (on-snow) variable (columns) used for testing hypothesis H1.

	**Total GRF estimation from acceleration of CoM**	
	**SI**	**JI**
CMJ max force (SI)	0.71, −0.05, 0.02	0.44, 0.25, 0.08
Knee flex MVC (SI)	0.78, −0.03, 0.01	0.79, 0.06, 0.01
Knee ext MVC (SI)	0.26, −0.18, 0.18	0.38, −0.34, 0.11
Hip ext MVC (SI)	0.81, −0.03, 0.01	0.17, 0.31, 0.29

**Adjusted p-value threshold was 0.0026. CMJ, countermovement jump; CoM, center of mass; GRF, ground reaction forces; JI, Jaccard index; MVC, maximal voluntary contraction; SI, symmetry index.**

**TABLE 4:** Triplets (*p*-value, coefficient in the linear regression model, *R*^2^) for each pair: independent (laboratory) variable (rows) and dependent (on-snow) variable (columns) used for testing hypothesis H2.

	**Inside leg total force pressure insoles**		**Outside leg total force pressure insoles**	
	**SI**	**JI**	**SI**	**JI**
CMJ max force (SI)	0.45, 0.53, 0.08	0.35, 2.20, 0.13	0.43, 0.46, 0.09	0.18, 2.93, 0.24
Knee flex MVC (SI)	0.11, 0.80, 0.32	0.16, 2.48, 0.26	0.93, −0.04, 0.00	0.58, 1.00, 0.05
Knee ext MVC (SI)	0.55, −0.50, 0.05	0.47, −2.04, 0.08	0.01, −1.46, 0.66	0.06, −4.65, 0.43
Hip ext MVC (SI)	0.02, 1.25, 0.63	0.04, 3.91, 0.53	0.37, 0.43, 0.13	0.09, 2.81, 0.41
Hip abd MVC (SI)	0.31, −0.69, 0.15	0.46, 1.73, 0.29	0.27, −0.62, 0.17	0.53, −1.43, 0.06
Trunk lat flex MVC (SI)	0.72, −0.15, 0.02	0.52, −0.89, 0.06	0.53, −0.21, 0.06	0.35, −1.24, 0.29

**Adjusted p-value threshold was 0.0004. CMJ, countermovement jump; JI, Jaccard index; MVC, maximal voluntary contraction; SI, symmetry index.**

**TABLE 5:** Triplets (*p*-value, coefficient in the linear regression model, *R*^2^) for each pair: independent (laboratory) variable (rows) and dependent (on-snow) variable (columns) used for testing hypothesis H3.

	**Inside leg entire foot pressure insoles measured force**				**Outside leg entire foot pressure insoles measured force**			
	**Backfoot**		**Forefoot**		**Backfoot**		**Forefoot**	
	**SI**	**JI**	**SI**	**JI**	**SI**	**JI**	**SI**	**JI**
Ankle flexion MVC (SI)	0.61, −0.79, 0.04	0.97, −0.09, 0.00	0.68, 0.44, 0.03	0.45, 1.80, 0.08	0.16, −1.00, 0.26	0.44, −1.14, 0.09	0.69, −0.35, 0.02	0.64, −1.04, 0.03
Ankle extension MVC (SI)	0.40, −1.90, 0.10	0.14, −4.87, 0.29	0.06, 2.61, 0.43	0.10, 2.26, 0.33	0.28, 1.17, 0.17	0.49, 1.51, 0.07	0.38, 1.09, 0.11	0.40, 2.71, 0.10

**Adjusted p-value threshold was 0.0021. JI, Jaccard index; MVC, maximal voluntary contraction; SI, symmetry index.*x*

## Discussion

The major finding is that asymmetries in GRFs during elite alpine skiing are not related to asymmetries in local muscular strength. More precisely, none of our three hypotheses was supported by the findings: none of the asymmetries between left and right turns in total GRF (H1) or in GRF acting on the inside and outside legs (H2) or rear- and forefoot GRF (H3) were associated with asymmetries in the parameters related to strength examined. Nevertheless, this group of elite slalom skiers exhibited significant inter-limb asymmetries in MVC during ankle flexion and hip extension, as well as in the GRFs on the inside leg while skiing.

The GRFs on the skier are highest during the steering phase and considerably lower during both the initiation and completion phases (Supej and Holmberg, 2010). Therefore, it is reasonable to assume that the most pronounced asymmetries in GRF caused by asymmetries in strength will occur during the steering phase. Therefore, the values of the dependent variables were computed for this phase only. The symmetry index (SI) utilized here is symmetrical [i.e., SI(L, R) = SI(R, L)], bounded by the interval [0,1], and increases monotonically as symmetry increases. This last characteristic is a requirement for meaningful interpretation of the results of linear multivariable regression models.

Since the absolute values for GRFs estimated using pressure insoles can deviate substantially from the true values (Lüthi et al., 2005; Stricker et al., 2010), acceleration of the CoM was also used here to compute the GRFs’ approximation. The most pronounced SI (∼98%), as well as the greatest JI (∼71%) for these absolute GRFs were relatively large compared to the other dependent variables examined. In practice, this means that the mean overall GRF on the skier during left and right turns is very similar, in spite of the other differences observed. This was probably due to the use of a highly accurate RTK GNSS to ensure that the course was set symmetrically, with the chosen slope having little or no tilting to either side. However, similarity in the absolute GRF during left and right turns does not necessarily mean that the skiers’ body movements were symmetrical.

Interestingly, as indicated by the values provided by the asymmetry in GRF indicated by the pressure insoles, the asymmetry in GRF was statistically significant for the inside leg only (*p* = 0.21 for the outside leg, Table 2). In other words, the force(s) acting on the right foot during right turns, when this leg was inside, was larger than the force(s) acting on the left leg, but this was not the case during left turns, when the right foot was outside. Although this difference might be related to the fact that five of our nine skiers exhibited right leg preference, the present findings cannot be used to fully support this possibility.

Although also not statistically significant, noticeably more maximal force was produced by the right leg during the countermovement jump (*p* = 0.12) (Table 1). Although vertical jump parameters were previously shown to be good predictors of slalom performance (Strojnik and Dolenc, 2009), here there was no association between performance and asymmetries associated with jumping and skiing. One speculative explanation for the lack of any such association is that the mean deviation from perfect symmetry with respect to the independent variables was no larger than ∼10%, in line with findings on elite Austrian alpine skiers (Steidl-Muller et al., 2018). Compared to the thresholds for safe return-to-sport proposed by Myer et al. (2006), our participants did not demonstrate levels of asymmetries as pronounced as, for example, those exhibited by skiers following reconstruction of the ACL. The recent observation that asymmetries in isometric leg extension strength constitute a significant risk factor for traumatic injury (Steidl-Muller et al., 2018) indicates that the only potential risk factor for some of our skiers could be their SI for MVC in connection with hip extension (89.5 ± 6.3%).

A previous investigation involving the same on-snow measurements as those employed here (but no strength tests) revealed that asymmetries in the GRF acting on the entire outside leg were associated with asymmetries in turning radius, which is related to performance (Supej et al., 2020). In the present case, the asymmetries in the production of skiing force (dependent variables) turned out to be greater than fundamental asymmetries (independent variables), a finding that is at least somewhat in agreement with earlier reports that fundamental motor asymmetries of approximately 10% lowered jump height and impaired change of direction by more than 10% (Hoffman et al., 2007; Bell et al., 2014). It can thus be speculated that asymmetries in strength might influence performance. Associations observed between asymmetries and performance appear to differ between sports and with respect to different measures of outcome (Bishop et al., 2018). Therefore, more specific and detailed characterization of potential relationships between asymmetries, with regards to both fundamental motor abilities and GRF during skiing, and the performance of elite alpine skiers would be of considerable value. Such an investigation should preferably involve a larger number of athletes who specialize in a variety of skiing disciplines, although this is difficult to arrange in the case of elite alpine skiing on-snow.

Another interesting observation here is that the SI for maximal power during the countermovement jump, as well as the corresponding indices for both maximal power and maximal force during the squat jump, exhibits exceptionally high symmetry (∼98%), with low standard deviations. Similarly, the mean SI for other fundamental movements were also relatively high, in general around 95%. The small sizes of these asymmetries and their highly limited variation may have obscured other relationships between fundamental and skiing-specific asymmetries, thereby leading to the rejection of all three hypotheses. It might be useful in a future study of this nature to include skiers with more pronounced asymmetries in physical abilities, perhaps even those who have “recovered” from an ACL injury, who, despite returning fully to competition, still exhibit asymmetries in inter-limb function and strength (Jordan et al., 2015a,b). This might reveal certain underlying relationships not present among uninjured elite skiers.

It is noteworthy that the symmetry values in skiing (dependent) variables indicated by their Jaccard indices (JI) were, in general, much lower that what the corresponding SI suggested (Table 2). This difference can probably be explained by the fact that the JI values reflect time-dependent behavior during the turning cycle, whereas the SIs describe the behavior on the basis of a single (usually mean) value associated with the turn. In practice, this means that the SI value for any given variable during skiing could be very high, while the corresponding JI is very low. Accordingly, the novel approach to analysis of asymmetries that we present here can provide new insights.

From a practical point of view, the relatively large asymmetries in skiing GRF indicated by the JI values (despite much smaller asymmetries as assessed on the basis of SI) suggest that, for example, the rate of force development and duration of the maximal GRF differ between left and right turns. The fact that elite skiers train extensively on snow, especially during the competition period (Gilgien et al., 2018), could contribute to the development of asymmetries in strength as the season progresses. Therefore, we suggest that testing regularly for asymmetries in combination with compensatory strength training, as required, can help avoid a potential increase in the risk of injury.

### Methodological Considerations

The current investigation encompassed one of the largest sets of full-body three-dimensional kinematic data on elite alpine skiers reported to date (in total, 720 turns), collected utilizing a state-of-the-art Global Navigation Satellite System (GNSS) and capture of inertial motion sensors with pressure insoles. Such measurement equipment has been shown to be reliable for use in field tests on alpine skiers (Krüger and Edelmann-Nusser, 2010; Supej, 2010; Spörri et al., 2015, 2016, 2018; Zorko et al., 2015; Falda-Buscaiot et al., 2017). In addition, our laboratory tests were performed with up-to-date, valid and reliable equipment. Nevertheless, a larger sample size would increase the power of the statistical tests, but it is extremely difficult to assemble a larger group of elite competitors to take measurements on-snow and maintain conditions on the slope for them that would allow objective analyses.

Nevertheless, this study did have certain limitations. The ski boot did not allow the sensors of inertial motion to be placed on the fore-foot, but since characterization of ankle mechanics was not part of our main goal, this did not affect the results.

As mentioned above, pressure insoles do not provide the most precise measurement of GRF during alpine skiing, giving 21 and 54% lower values than those obtained with dynamometers for the outside and inside skis, respectively (Stricker et al., 2010). At the same time, pressure insoles can monitor pressure on different parts of the foot (e.g., the fore- and rear-foot) and the right and left legs separately. Moreover, the type and extent of bias are likely to be similar for the left and right legs, so that values provided by pressure insoles can be helpful in revealing differences between the legs, which was the primary focus here.

Finally, since our measurements were performed on a moderate incline under nearly ideal conditions with respect to snow and weather, generalization of the findings is not straightforward. Future studies on slopes with different inclines and with different gate set-ups and snow conditions are warranted. Moreover, a comparison of left and right turns on a course that tilts to the side would be of interest, although this would require different methodological approaches and data processing.

## Conclusion

Ours is the first examination of potential relationships between asymmetries in fundamental muscular strength and asymmetries exhibited on-snow by elite alpine skiers. Despite the well-known importance of physical fitness in this context (Strojnik and Dolenc, 2009) and the association of GRF with parameters related to performance (Supej et al., 2020), our present findings do not indicate that muscular strength exerts a direct impact on GRFs. However, the asymmetries in muscular strength among the skiers involved here were probably too small to exert an observable impact on GRF asymmetries in connection with such a complex sport as slalom skiing. Thus, the potential existence of such relationships remains to be elucidated, perhaps using athletes who demonstrate more pronounced asymmetries in strength. In addition, it might be revealing to examine whether fatigue augments skiing asymmetries and, if so, whether this augmentation is more closely related to asymmetries in basic strength.

## Data Availability Statement

The raw data supporting the conclusions of this article will be made available by the authors, without undue reservation.

## Ethics Statement

The studies involving human participants were reviewed and approved by National Medical Ethics Committee. The patients/participants provided their written informed consent to participate in this study.

## Author Contributions

MS and H-CH designed the study. MS, NŠ, JO, UK, and MM prepared the equipment for data collection, performed the measurements, and prepared the platform for computations. JO performed data pre-processing, processing, and statistical analysis. JO, NŠ, and MS performed the data analysis and interpretation. JO, MS, NŠ, and H-CH contributed to drafting the manuscript. All authors provided feedback on and approved the final version of the manuscript.

## Conflict of Interest

NŠ was partially employed by the company S2P, Science to Practice, Ltd. The remaining authors declare that the research was conducted in the absence of any commercial or financial relationships that could be construed as a potential conflict of interest.
