# Supplementary material for: Asymmetries in Ground Reaction Forces During Turns by Elite Slalom Alpine Skiers Are Not Related to Asymmetries in Muscular Strength
Source: Front Physiol. 2021 Mar 30;12:577698. doi: 10.3389/fphys.2021.577698 (PMC8042208; doi:10.3389/fphys.2021.577698)
Supplement: Supplementary file 1 [file Presentation_1.pptx]

## Slide 1
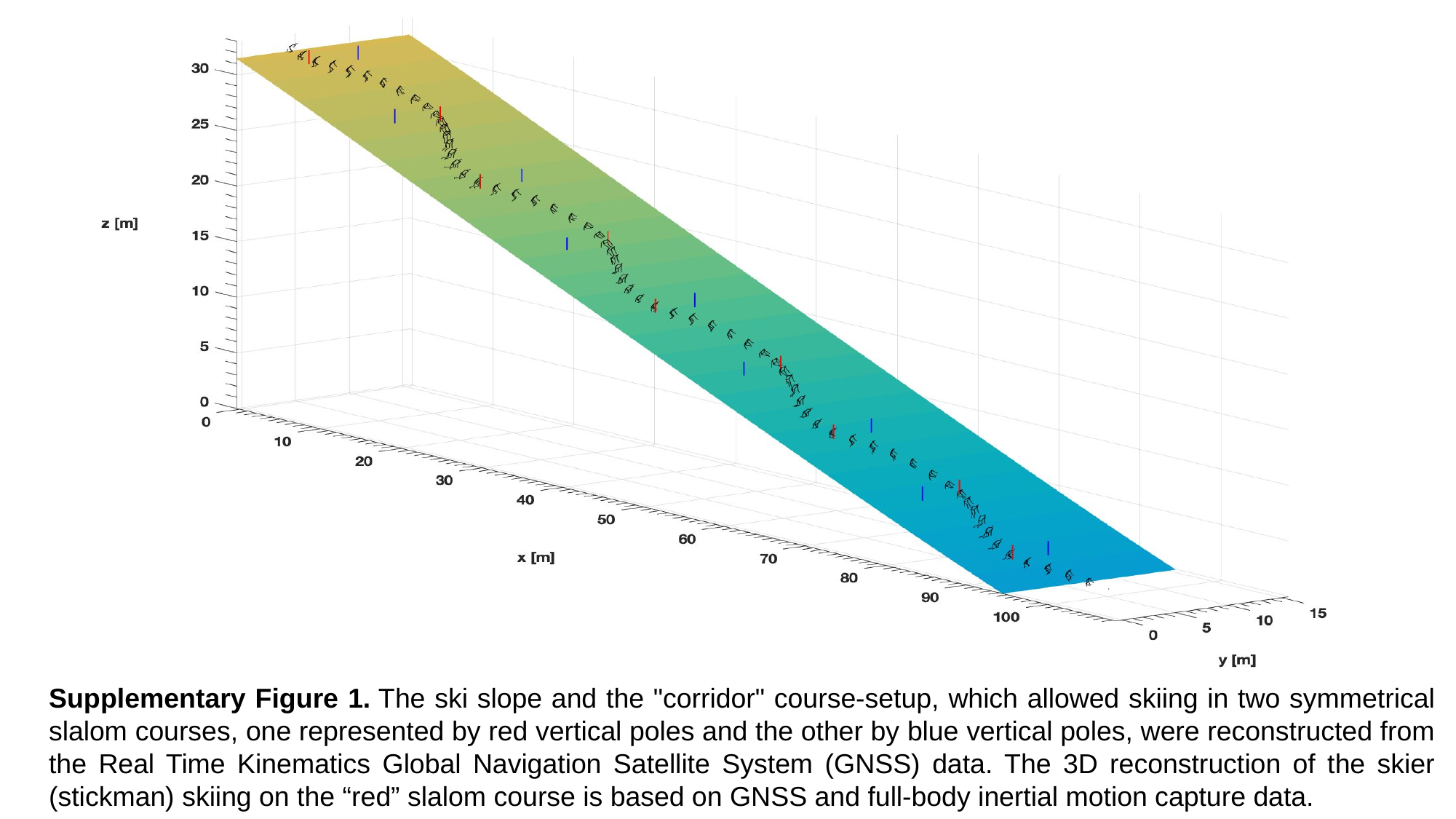

Supplementary Figure 1. The ski slope and the "corridor" course-setup, which allowed skiing in two symmetrical slalom courses, one represented by red vertical poles and the other by blue vertical poles, were reconstructed from the Real Time Kinematics Global Navigation Satellite System (GNSS) data. The 3D reconstruction of the skier (stickman) skiing on the “red” slalom course is based on GNSS and full-body inertial motion capture data.
